# Implanting CRT via Endo-Cardiac Route When Tricuspid Valve is Metallic

**Published:** 2014-01-01

**Authors:** Mohammad Vahid Jorat, Amir Aslani, Mohammad Hossein Nikoo

**Affiliations:** 1 Cardiovascular Research Center, Shiraz University of Medical Sciences, Shiraz, IR Iran

**Keywords:** CRT, Tricuspid Valve, Heart Failure

## Abstract

Prosthetic tricuspid valve is an obstacle to implant cardiac devise. Cardiac Resynchronization therapy is one of the most popular therapies for heart failure patients these days. We present this case of prosthetic tricuspid valve and left ventricular dysfunction which we overcome the problem by implanting two leads to coronary sinus branches. Patient improved in few months of follow up.

## 1. Introduction

Heart failure is prevalent and its prevalence rate is following an increasing trend (1). Cardiac Resynchronization Therapy (CRT) is beneficial for about 25% of those with left bundle branch in ECG, lower than 35% ejection fraction, and moderate to severe dyspnea on full medical therapy without correctable cause (2).

Implanting CRT includes one lead to the right ventricle, one lead to the left ventricle, and the 3rd to the right atrial appendage. We implant LV lead routinely via the coronary sinus. LV access in open-heart surgery is another option. Few implants from septostomy have also been reported (3). Metallic tricuspid valve is an obstacle to enter the right ventricle causing the researchers to have dilemma when implanting CRT through open-heart surgery. In this study, we present one of these patients and report our attempt to avoid open-heart surgery by implanting two LV leads from the coronary sinus (4).

## 2. Case Presentation

A 52 year old man with severe dyspnea on exertion came to our attention. He was unable to do his job and had difficulty in doing his grooming. The patient’s past medical history showed aortic valve replacement 20 years ago. Another valve surgery had also occurred 7 years ago during which, aortic, mitral, and tricuspid valves were replaced by metallic valves.

Echocardiography showed normal functioning prosthetic valves, but EF = 30%, EDD = 87, and ESD = 65.

In addition, ECG revealed wide QRS and prolonged PR.

During ambulatory ECG monitoring, some brief episodes of atrial tachycardia were also noted. However, coronary angiography revealed normal findings.

The two previous valve surgeries would make the 3rd one a difficult operation due to severe adhesions; therefore, we decide to implant two LV leads from CS. After the implantation, the patient’s dyspnea improved. Then, we started Amiodarone to prevent atrial tachycardia and also increased carvediolol.

**Figure 1.**
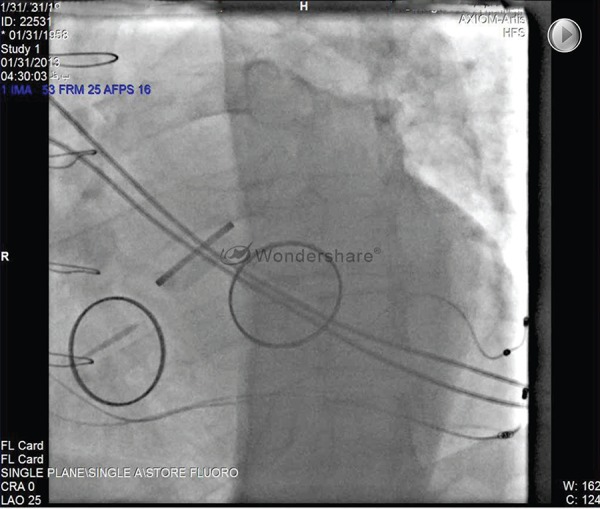
LAO Projection Showed 3 Prosthetic Valves and Position of 2 LV Leads in the Lateral Veins

**Figure 2.**
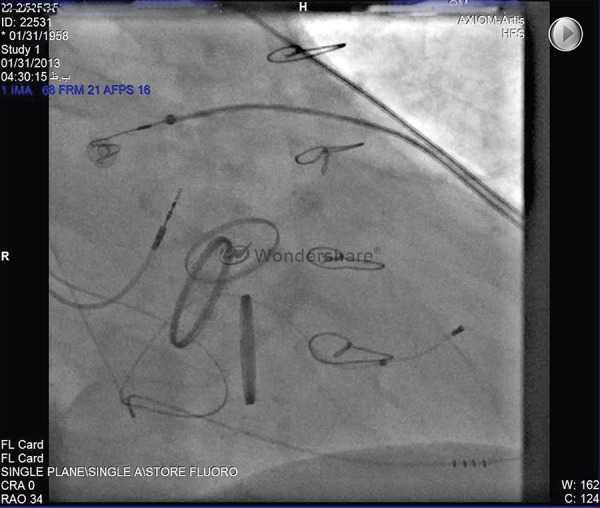
RAO Projection Showed Final 3 Prosthetic Valves and 3 Leads of CRT

**Figure 3.**
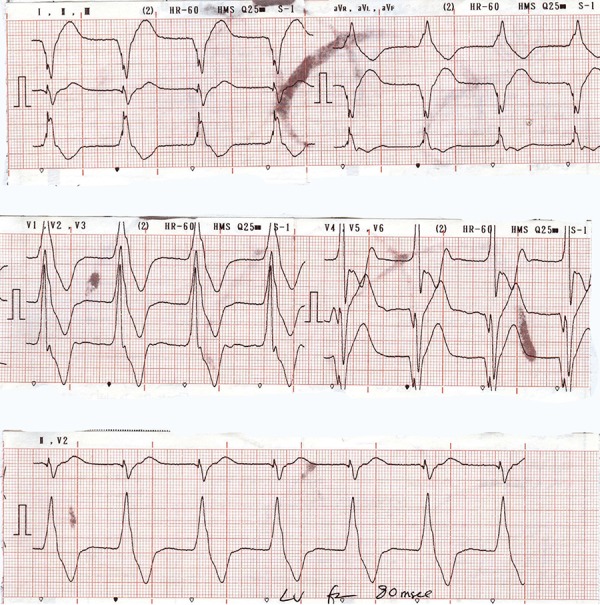
ECG after CRT Implantation

ECG and the fluoroscopic image are presented in Figures 1, 2, and 3.

## 3. Discussion

Although CRT implantation alleviate symptom and improve outcome in heart failure patients, there are some challenging cases sometimes. Implanting any device in patients with prosthetic tricuspid valve is challenging because RV. is out of reach.

Implanting leads to left ventricle epicardium from coronary sinus route is possible and promising in this group (4).

Coronary Sinus is a way to reach ventricles in patients with prosthetic Tricuspid Valve. This way permits CRT implantation in this group without hazard of open-heart surgery.
